# Tumor development in patients with neurofibromatosis type 1: A retrospective cohort study including 644 patients

**DOI:** 10.1016/j.jdin.2024.03.009

**Published:** 2024-04-20

**Authors:** Kaya L. Curtis, Samantha Jo Albucker, Victoria De Barros, Yuqing Qiu, Shari R. Lipner

**Affiliations:** a Weill Cornell Medical College, New York, New York; b Tulane University School of Medicine, New Orleans, Louisiana; c Department of Population Health Sciences, Weill Cornell Medicine, New York, New York; d Department of Dermatology, Weill Cornell Medicine, New York, New York

**Keywords:** basal cell carcinoma, cancer, glomus tumor, malignancy, melanoma, neoplasia, neurofibromatosis type 1

*To the Editor:* Neurofibromatosis type 1 (NF1) is an autosomal dominant cancer predisposition syndrome due to loss-of-function mutations in the tumor suppressor gene *NF1*. Patients with NF1 have 5% to 15% higher risk of neoplasm development than the general population, with earlier age of onset.^1^ Since NF1-associated neoplasm prevalence remains understudied, we conducted a retrospective study assessing prevalence of NF-1 associated benign and malignant nonneurofibroma tumors.

Medical records of patients with NF1 at Weill Cornell Medicine 1979 to 2022 were reviewed for neoplasia diagnosis (Supplementary Methods, available via Mendeley at https://data.mendeley.com/datasets/mfwmm87m7r/1).

Six hundred forty-four patients with NF1 were analyzed, with mean age 29 years (range 3-86) (Table I). Overall, 28.1% developed a nonneurofibroma neoplasm, of which 23.0% and 5.1% had single and ≥1 neoplasms, respectively (Table II). Most common neoplasms were glioma (22.6%) and malignant peripheral nerve sheath tumor (4.5%).

**Table I:** Demographic characteristics of patients with neurofibromatosis type 1

Patient characteristics	Patients with neurofibromatosis type 1
Age (mean years; min-max)	29 (3-86)
Age at NF1 diagnosis (mean years; min-max)	4 (0-54)
Sex (%, *n*)	
Female	52.3% (337)
Male	47.7% (307)
Race (%, *n*)	
White	39.6% (255)
Black	6.8% (44)
Asian	3.9% (25)
Native American or Alaska Nation	0.3% (2)
Other combinations not described	15.8% (102)
Race not reported	33.5% (216)
Ethnicity (%, *n*)	
Hispanic	11.3% (73)
Not Hispanic	46.1% (297)
Ethnicity not reported	42.5% (274)

*NF1*, Neurofibromatosis type 1.

**Table II:** Nonneurofibroma neoplasms in patients with neurofibromatosis type 1

Neoplasm type	Prevalence, no. (%)	Age at diagnosis, mean (range)
Nonneurofibroma neoplasms	181 (28.1%)	NA
Single neoplasm	148 (23.0%)	NA
Multiple neoplasms	33 (5.1%)	NA
Glioma		
Low grade	144 (22.3%)	8.6 (0-52)
Optic pathway glioma	106 (16.5%)	6.3 (0-52)
Nonoptic pathway glioma	38 (5.9%)	15.2 (3-40)
High grade	2 (0.3%)	35.5 (9-62)
Sarcoma		
Malignant peripheral nerve sheath tumor	29 (4.5%)	32.4 (2-78)
Gastrointestinal stromal tumor	4 (0.6%)	46.0 (33-55)
Angiosarcoma	1 (0.2%)	16.0
Spindle cell sarcoma	1 (0.2%)	Unknown
Leiomyosarcoma	1 (0.2%)	64.0
Breast cancer	12 (1.9%)	41.9 (31-52)
Endocrine neoplasia		
Pheochromocytoma	3 (0.5%)	40.0 (36-41)
Neuroendocrine tumor	3 (0.5%)	46.0 (28-66)
Follicular thyroid carcinoma	1 (0.2%)	45.0
Skin cancer		
Basal cell carcinoma	4 (0.6%)	40.0 (29-58)
Leukemia		
Acute lymphoblastic leukemia	1 (0.2%)	42.0
Chronic lymphocytic leukemia	1 (0.2%)	81.0
Genitourinary neoplasia		
Prostate	2 (0.3%)	65.0 (62-68)
Bladder	2 (0.3%)	59.0 (54-64)
Lymphoma		
Follicular lymphoma	1 (0.2%)	47.0
Other		
Lung	3 (0.5%)	47.7 (35-54)
Meningioma	1 (0.2%)	Unknown
Glomus tumor	1 (0.2%)	52.0
Parotid oncocytoma	1 (0.2%)	Unknown
Essential thrombocytosis	1 (0.2%)	64.0

Most frequent nonneurologic neoplasms included breast (1.9%), basal cell carcinoma (0.6%), gastrointestinal stromal tumor (0.6%), and pheochromocytoma (0.5%), with mean age at diagnosis 42, 40, 46, and 40 years, respectively. There were no melanoma or squamous cell carcinoma cases. There was 1 glomus tumor (0.2%) and 2 suspected glomus tumors (fingertip pain).

Our study is one of few large-scale studies examining prevalence of a broad range of NF1-associated neoplasia. Our findings were similar to Landry et al’s retrospective cohort study^1^ of 1607 patients with NF1 (median age 19 years), with the exception of their higher prevalence of malignant peripheral nerve sheath tumor (15.1%), which may be due to sampling bias.

In our study, basal cell carcinoma was rare, and no patients were diagnosed with squamous cell carcinoma or melanoma, similar to Landry et al^1^ reporting nonmelanoma and melanoma prevalence 0.9% each. Trinh et al’s retrospective case control study^2^ of 4122 patients with NF1 (mean age 47 years) found basal cell carcinoma, squamous cell carcinoma, and melanoma prevalence of 4.3%, 2.6%, and 1.8% respectively, with increased melanoma (odds ratio, 2.27; 95% CI, 1.75-2.93) and keratinocyte carcinoma risk (odds ratio, 1.31; 95% CI, 1.15-1.51). Comparatively lower skin cancer prevalence in our and Landry’s studies^2^ may be due to young age.

Our cohort only had maximum 3 glomus tumors. In 2 case series^3^^,^^4^ of 42 and 21 histopathologically confirmed glomus tumors, 17.7% (6/34 patients) and 14.3% (3/21) were in patients with NF1 (mean age 45 years both), respectively. Lower glomus tumor prevalence in our cohort may be due to glomus tumor underdiagnosis.

Our study is limited by retrospective single-center design. Clinical detail per case was heterogeneously reported. Not all patients had long term follow-up.

Our study highlights the importance of awareness of malignancy screening guidelines for patients with NF1, including breast cancer screening beginning at age 30 in women, biochemical testing for pheochromocytoma and paraganglioma in the setting of unexplained high blood pressure, and regional magnetic resonance imaging for malignant peripheral nerve sheath tumor in the setting of new pain or motor deficit.^5^

Our cohort study confirms prevalence and broad range of nonneurofibroma neoplasms in patients with NF1, underscoring importance of counseling and regular screening. Glomus tumor and skin cancer prevalence was surprisingly low, likely due to underdiagnosis and young age. Prospective studies with age stratification are needed to assess risk of NF-1 associated glomus tumors.

## Conflicts of interest

Dr Lipner has served as a consultant for Ortho-Dermatologics, Eli Lilly, BelleTorus Corporation, and Moberg Pharmaceuticals. Authors Curtis, Albucker, De Barros, and Qiu have no conflicts of interest to declare.
